# The Troy Microneedle: A Rapidly Separating, Dissolving Microneedle Formed by Cyclic Contact and Drying on the Pillar (CCDP)

**DOI:** 10.1371/journal.pone.0136513

**Published:** 2015-08-26

**Authors:** Miroo Kim, Huisuk Yang, Suyong Kim, Chisong Lee, Hyungil Jung

**Affiliations:** Department of Biotechnology, Yonsei University, Seoul, Republic of Korea; University College Cork, IRELAND

## Abstract

In dissolving microneedle (DMN)-mediated therapy, complete and rapid delivery of DMNs is critical for the desired efficacy. Traditional patch-based DMN delivery, however, may fail due to incomplete delivery from insufficient skin insertion or rapid separation of microneedles due to their strong bond to the backing film. Here, we introduce the Troy microneedle, which was created by cyclic contact and drying on the pillar (CCDP), and which enabled simultaneous complete and rapid delivery of DMN. This CCDP process could be flexibly repeated to achieve a specific desired drug dose in a DMN. We evaluated DMN separation using agarose gel, and the Troy microneedle achieved more complete and rapid separation than other, more deeply dipped DMN, primarily because of the Troy’s minimal junction between the DMN and pillar. When Troy microneedles were applied to pig cadaver skin, it took only 15 s for over 90% of encapsulated rhodamine B to be delivered, compared to 2 h with application of a traditional DMN patch. *In vivo* skin penetration studies demonstrated rapid DMN-separation of Troy microneedles still in solid form before dissolution. The Troy microneedle overcomes critical issues associated with the low penetration efficiency of flat patch-based DMN and provides an innovative route for DMN-mediated therapy, combining patient convenience with the desire drug efficacy.

## Introduction

A dissolving microneedle (DMN) is a micro-sized needle made of biodegradable polymer that encapsulates pharmaceuticals within a matrix and releases the drugs after skin insertion via dissolving of the polymeric compound [1, 2]. DMN has received attention as an innovative transdermal drug delivery system due to minimum pain on delivery, biocompatibility and patient convenience [3, 4]. In spite of these advantages, some challenges for the complete delivery of encapsulated pharmaceuticals remain because of incomplete insertion of the DMNs. Usually, DMNs are fabricated on a sticky flat patch that facilitates their insertion into the skin and keeps them on the skin surface until they are completely dissolved. Due to the viscoelastic properties of skin, however, DMNs on a flat patch often do not completely penetrate the skin, even with a strong enough insertion force, complicating delivery of the full amount of encapsulated drugs [5, 6, 7]. Patch-based DMNs must be left in place for a long time to allow for full dissolution of the polymer matrix in the skin, which may generate an allergic response in some patients [8]. For these reasons, a better DMN delivery system with complete insertion and rapid administration is needed.

Several approaches have attempted to overcome the limitations of traditional DMN patches. Two-layered DMNs that only encapsulate drugs at the tip have been introduced to overcome incomplete drug delivery [9]. Even with incomplete DMN insertion, this two-layered system can deliver all encapsulated drugs without a loss. Alternatively, a system where DMNs are attached to a water soluble backing film has been developed to deliver the DMNs by dissolving the water soluble film [10]. In this system, water is used as an dissolving accelerator, allowing drugs in the DMNs to be delivered rapidly (within 10 min). This new DMN formation also depends on a flat backing film, however, and so complete insertion is needed. In addition, the water used to dissolve the patch may also dissolve the portion of the DMNs not completely inserted into the skin.

A sticky patch would not be necessary if the DMNs could be rapidly separated from the base and delivered into the skin before completely dissolution. Patchless DMN delivery requires another substrate that can provide sufficient mechanical insertion force to overcome the skin's viscoelasticity. Arrowhead and embeddable chitosan DMNs address incomplete insertion issues with micro-sized, protruded supporting arrays that allow for easy insertion [11, 12]. Despite their superior penetration efficiency, however, fast DMN delivery remains a challenge. DMNs on supporting arrays are mostly fabricated by micro-molding, and complete removal of the DMN arrays from the PDMS mold is accomplished by peeling. During this process, the tips of the supporting array have to be physically anchored to the inner bases of DMNs (over 100 μm piercing depths), creating an unavoidable vertical overlap that acts as a barrier to fast delivery. To the best of our knowledge, no DMN delivery system has been developed that satisfies the requirement for both complete insertion and rapid administration.

In this study, we introduce the Troy microneedle (Troy MN), which uses cyclic contact and drying on the pillar (CCDP) to facilitate complete insertion of DMNs into the skin. Easy permeation of interstitial fluid between the DMNs and pillars causes rapid separation following complete insertion, even if the DMNs remain undissolved. The term “Troy” is from story of Trojan horse in Greek mythology, which has similar traits, insertion and separation steps, with our product. This novel, patchless DMN delivery system overcomes critical challenges associated with the low penetration efficiency and impeded drug delivery of flat patch-based DMN systems, showing promise for widespread application in the intradermal delivery of many pharmaceuticals with complete and rapid administration.

## Materials and Methods

### Pillars

Blunt pillars were achieved by cutting sharp tipped-metal microneedles (Goryeo Sujichim, Seoul, Korea) with a laser system (K2 Laser System, Gyeonggi-do, Korea). The pillars were 170 ± 5 μm in diameter and 800 ± 35 μm long. Individual pillars were assembled to a homemade applicator through holes to prepare a pillar array with 600 μm protrusion from the applicator. Distance between pillars was 2.0 mm.

### Polymer solutions

Polyvinylpyrrolidone (PVP, 360 kDa, Sigma, St Louis, MO, USA) and Chitosan (50~190 kDa, Sigma, St Louis, MO, USA) were used as biodegradable polymers to fabricate DMNs. The hydrophilic fluorescent dye Rhodamine B (479 Da, Sigma, St Louis, MO, USA) was used as model drug. Rhodamine B and polymers were dissolved in deionized water, homogenized and degassed simultaneously with a planetary centrifugal mixer (ARV-310, THINKY Corporation, Tokyo, Japan), and used as polymer solutions.

### Troy MN fabrication via CCDP

Drug encapsulation step: After oxygen plasma treatment for 5 min to increase pillar hydrophilicity, the pillar tips were dipped into the PVP solution (30%, w/v) to a depth of 200 ± 50 μm. Pillars were lifted out of the solution and dried by symmetric air blowing (0.15 kg∙f/cm, filtrated by WS-500, GyeSeong Science, Gyeonggi-do, Korea) for 10 s to produce a thin polymer film on the pillar tip surface. Then, pillars were contacted to the polymer solution mixed with rhodamine B and dried again by symmetric air blowing after lifting from the polymer solution. Repeated contact and drying cycles resulted in a spherical polymer structure on the tip of each pillar. The down and up speeds were set to 1.0 mm/min, and 10 s of air blowing was applied in the drying step.

Microneedle fabrication: Pillars with spherical polymers were dipped into PVP solution (45%, w/v) without dye and lifted up immediately. The viscous PVP solution covering the spherical structure was shaped into a microneedle by the drawing lithography method [13–15]. Briefly, viscous PVP was contacted to carboxymethylcellulose (CMC, 90 kDa, Low-viscosity, St Louis, MO, Sigma) film coated onto a metal plate in advance, then lifted to produce a microneedle shape by controlled drawing and air blowing for four min at room temperature. Because the polymer was left on the lower plate in this separation step, the second polymer was used without drug to prevent drug loss in this final microneedle fabrication step. Fabricated Troy MNs were isolated from the bottom plate.

### Dose control through concentration of the PVP solution

Polymer solutions were made by mixing rhodamine B (1%, w/v) with a 10, 20, 30 or 40% (w/v) PVP solution. The spherical micro-structure was fabricated by repeated contact and drying cycles 1, 3, 5 or 7 times. To analyze the amount of rhodamine B in each spherical micro-structure, dried spherical structures (4 × 4) were dissolved in 1 ml deionized water for each experimental group and analyzed by fluorescence spectrometer (LS55, Perkin-Elmer, Shelton, CT, USA) at the appropriate excitation (553 nm) and emission (627 nm) wavelengths.

### Evaluation of skin insertion frequency

Array of DMNs on pillars (5 × 5) was fabricated by different numbers of contact and drying cycles (1, 3, 5, 7 and 9) using 30% (w/v) PVP solution. Fabricated DMN arrays were applied to pig cadaver dorsal skin (1.5 ± 0.2 mm thick and 2.5 × 2.5 cm^2^ in size), and skin insertion frequency was evaluated by counting successfully penetrated DMNs after application. Experiment was conducted for four times under the same condition.

### Live detection of DMN-separation in agarose gel

Agarose gel (2.0% w/v, molecular biology grade, Invitrogen, France) was used for live imaging of DMN-separation from the pillar array. DMNs were fabricated using chitosan (7%, w/v) mixed with rhodamine B (1%, w/v) as a polymer solution using homemade pillars with different diameters (170, 350 and 500 μm). Coating depth on the side walls of the pillars was set to 30 ± 5 (where the pillar just contacted the surface of the polymer solution), 200 ± 25 or 400 ± 40 μm, and the number of contact (or dipping) and drying cycles was set to 5 or 8 times. Fabricated DMNs (1 × 3) were penetrated into the agarose gel and removed after 5, 10, 20, 30, 40, 50, and 60 s. DMN-separation images were taken simultaneously with a microscope (Samwon Science Inc., Seoul, Korea). All tests were repeated four times under the same conditions.

### DMN patch

DMN patches were fabricated by droplet-born air blowing (DAB) method with a two-layered system [15]. PVP (30%, w/v) solution with rhodamine B (1%, w/v) was used as the DMN backbone material and carboxymethylcellulose (CMC, 90 kDa, Low-viscosity, Sigma, St Louis, MO, USA) solution was used as backing film with a thickness of 80 ± 5 μm. PVP solution (30%, w/v) without dye was used for the base structure formation, and 0.5 μL of rhodamine B-dissolved viscous PVP solution was dispensed on the PVP base structures at plates by dispenser (ML-5000X, Musashi, Japan). After the dispensed droplets at lower and upper plates were contacted, upper plate was lifted up at a speed of 1.5 mm/min, and then dried by air blowing (0.15 kg∙f/cm, filtrated by WS-500, GyeSeong Science, Gyeonggi-do, Korea) for 6 min at room temperature. Fabricated DMNs were finally isolated from each surface at a speed of 30 mm/min. The diameter and height of the DMN base structures were approximately 700 and 150 μm, respectively, and the length and tip diameter of the DMNs were 600 ± 30 and 35 ± 5 μm, respectively (S1 Fig).

### Minimum application time analysis

Troy MN and DMN patches (each 5 × 5) with 10 μg of encapsulated rhodamine B were applied to full thickness pig cadaver dorsal skin (1.5 ± 0.2 mm thick and 2.5 × 2.5 cm^2^ in size). After 5 to 100 s and 10 to 120 min application for the Troy MNs and DMN patches, respectively, the amount of rhodamine B remaining on the pillars and backing film was analyzed by fluorescence spectrometer.

### *In vitro* skin permeation study

Troy MNs and DMN patches (each 5 × 5) with 10 μg of encapsulated rhodamine B were applied to pig cadaver dorsal skin (1.5 ± 0.2 mm thick and 2.5 × 2.5 cm^2^ in size) for 30 s and 100 min, respectively. A Franz diffusion cell chamber filled with 7 ml of pH 7.4 phosphate buffered (PBS) was stirred continuously at 700 rpm and the temperature was maintained at 37 ± 0.5°C by a thermostatic water pump (AND Korea, Anyang-si, Korea). The receptor medium (1 ml) was withdrawn at 30 min and every hour up to 9 h after application. After 9 h, the skin was cut into small pieces and stored in 1 ml of methanol overnight to extract any deposited rhodamine B from the skin. The amount of rhodamine B was measured by fluorescence spectrometer.

### *In vivo* skin penetration study

Animal experiment was approved by the Institutional Animal Care and Use Committees of Avison Biomedical Research Center (IACUC-2013-0207-3). Intraperitoneal injection of Zoletil 50 (35 mg/kg) and Rompun (2 mg/kg) was carried out for anesthesizing the animals. After ending the experiment, carbon dioxide (CO_2_) was used for sacrificing. Female Sprague-Dawley rats (8 weeks, Orient Bio Inc., Seoul, Korea) were anesthetized and their dorsal hair was trimmed with an electric shaver to leave approximately 1.0 mm of hair. The application site was cleaned with 70% ethanol. Troy MNs (5 × 5) were assembled in a homemade applicator and applied to the rat dorsal skin vertically by hand for 30 s. After the applicator was removed, the region was washed carefully with wet tissue. Right after CO_2_ euthanasia, the target region was removed by scissors and keep in a—60°C before biopsy. Frozen tissue was sliced vertically with a microtome blade (Edge-Rite, Thermo Scientific, Waltham, MA, USA) and sections were examined by microscopy.

### Statistical analysis

Every experiment was performed at least three times under same conditions to enhance accuracy of result. Error bars in graphs represent standard error of mean (s.e.m.) or standard deviation (s.d.). SPSS Statistics 21 software was used to analyze data, based on analysis of variance (ANOVA).

## Results and Discussion

### Two compartmental fabrication steps of CCDP

**Fig 1:**
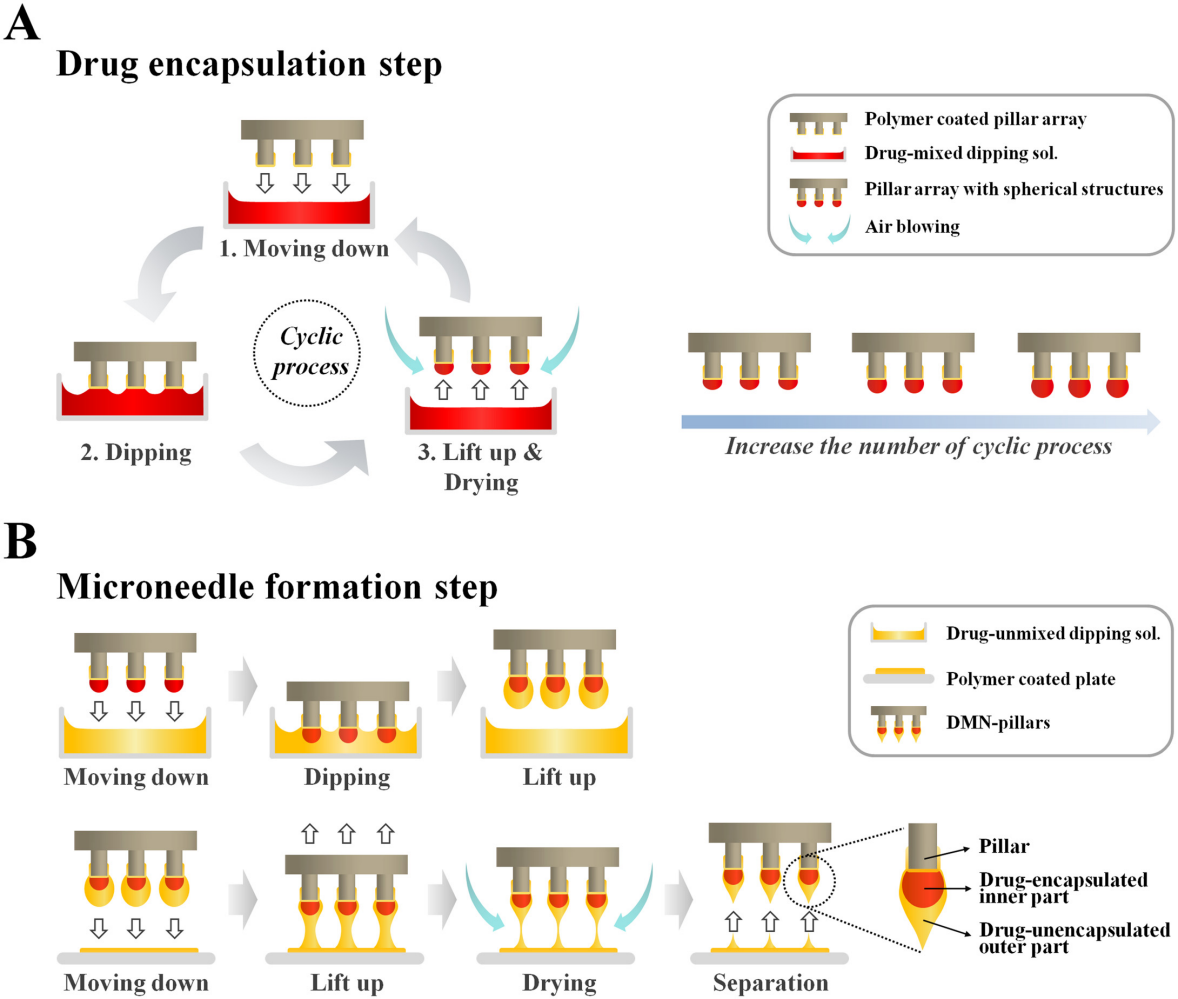
Schematic illustration of the CCDP process. The total process consisted of two independent steps. (A) In the first step (drug encapsulation), pillars were contacted with a mixed drug-polymer solution and then lifted and dried with air blowing. This contact and drying cycle was repeated to optimize drug encapsulation, producing spherical structures on each pillar. (B) In the second step (microneedle formation), polymer solution without drug was used to prevent loss of drug during DMN fabrication, resulting in sharp tipped-DMNs on the end surface of the pillars.

As shown in Fig 1, the CCDP process consisted of two independent steps: drug encapsulation for optimal drug dose control and microneedle formation to control the strength and sharpness of the DMNs. The drug encapsulation step fabricated spherical structures containing drug on the end surface of pillars (Fig 1A). Once the pillars made contact with the surface of a polymer solution containing the target drug, the viscous solution was drawn onto the polymer film on the tip of pillar due to the cohesive properties of polymer molecules. Because the ultimate purpose of CCDP was to fabricate DMNs on the end surface of the pillars, deep dipping (over 100 μm), which produced polymer coating on pillar side walls, was minimized (data not shown). After pillars contacted the polymer solution, they were lifted up and dried by air blowing for 10 s. In this drug encapsulation step, the polymer solution conformed to the spherical structure on the pillar, increasing the volume of the sphere as the contact and drying cycle was repeated, allowing the optimal drug dose to be encapsulated in the structure.

In the subsequent microneedle formation step, microneedle structures were produced on the drug-encapsulated spherical polymer structures (Fig 1B) using polymer solution without drug. The dipping depth was controlled to prevent excess at the junction between the spherical structure and the end surface of each pillar. After lifting the pillars from the second polymer solution, the pillar arrays were contacted to a flat surface without drying. Microneedles were formed by controlled drawing of the liquid form of the second polymer [15]. The resulting DMNs were fabricated on the end surface of the pillars and contained drugs encapsulated only in their inner layers.

### Drug dose control via CCDP

**Fig 2:**
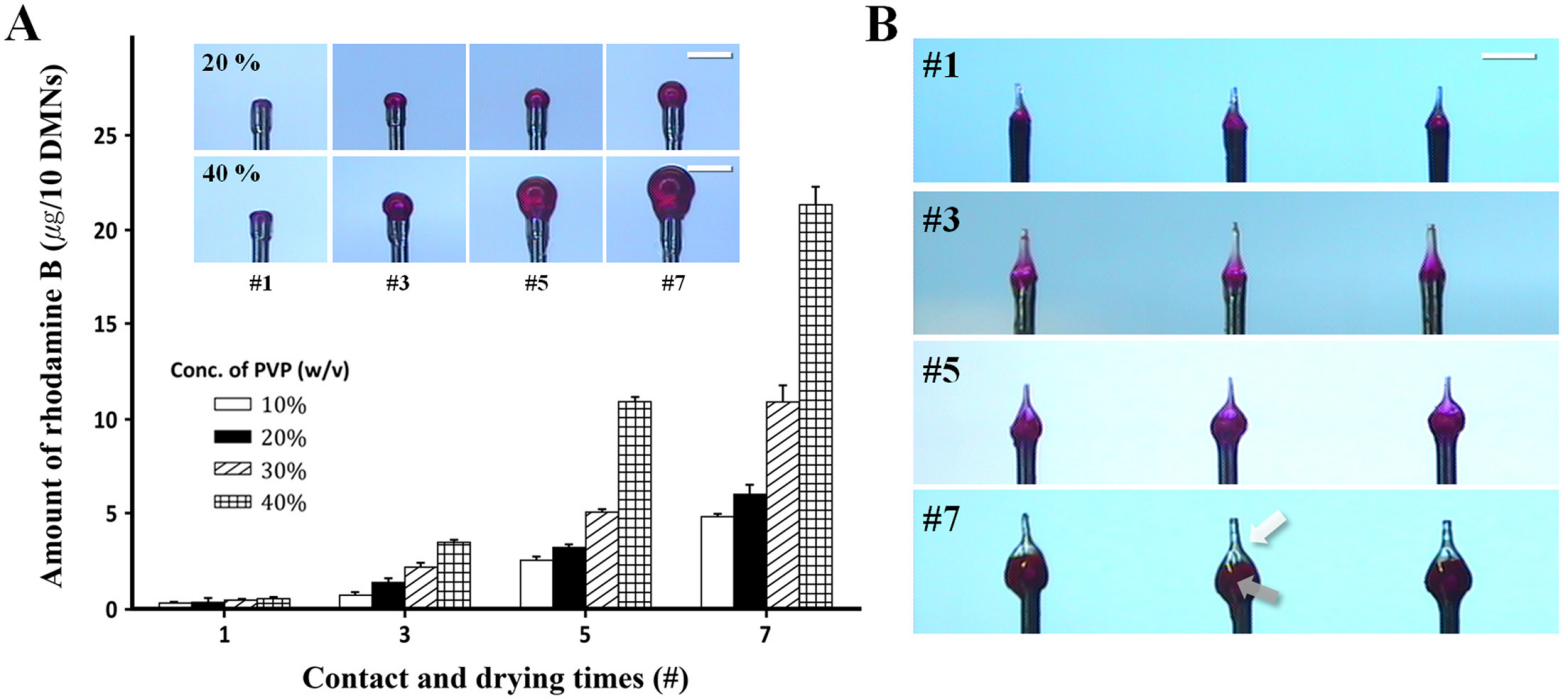
Drug dose control via contact and drying cycle. (A) The amount of rhodamine B rose exponentially as the number of contact and drying cycles and PVP concentration increased (n = 3). Data are shown as mean ± s.e.m. Images indicate that rhodamine B-encapsulated spherical structures created through contact and drying cycles increased when using 20 (upper) and 40% (bottom) PVP solution. (B) After the microneedle formation step, Troy MNs were fabricated as sharp tips without drug (white arrow) over inner spherical structures containing drug (gray arrow). Scale bars, 500 μm.

As shown in Fig 2A, the amount of encapsulated rhodamine B in the spherical structures increased exponentially as the number of contact and drying cycles increased for all tested concentrations of PVP. Because a greater amount of viscous solution was drawn onto the surface of the spherical structure with each cycle, the encapsulated rhodamine B increased exponentially (p < 0.0001 for all tested concentrations of PVP). The amount of encapsulated rhodamine B also rose as the concentration of PVP increased from 10 to 40% when cycle number was held constant (p < 0.0001 for all tested cycle number). The amount of rhodamine B encapsulated by 5 cycles using a 40% (w/v) PVP solution was similar to that of 7 cycles using a 30% (w/v) PVP solution (Fig 2A), indicating that fewer cycles are needed with higher concentrations of polymer to encapsulate the same amount of drug. In addition, a lower concentration of polymer could be used to encapsulate the desired amount of drug, as long as the number of contact and drying cycles is increased.

Fig 2B shows the final product of DMNs formed by CCDP after the microneedle formation step with sharp tips on the spherical structures. The total length of the DMNs and the maximum axial diameter increased from 380 ± 25 to 750 ± 25 μm and from 220 ± 9 μm to 430 ± 22 μm, respectively, as the number of dipping and drying cycles increased from 1 to 7. The diameter of the DMN tips, on the other hand, was stable at 45 ± 10 μm regardless of the volume of the spherical structures (S1 Table).

### Evaluation of skin insertion frequency

**Fig 3:**
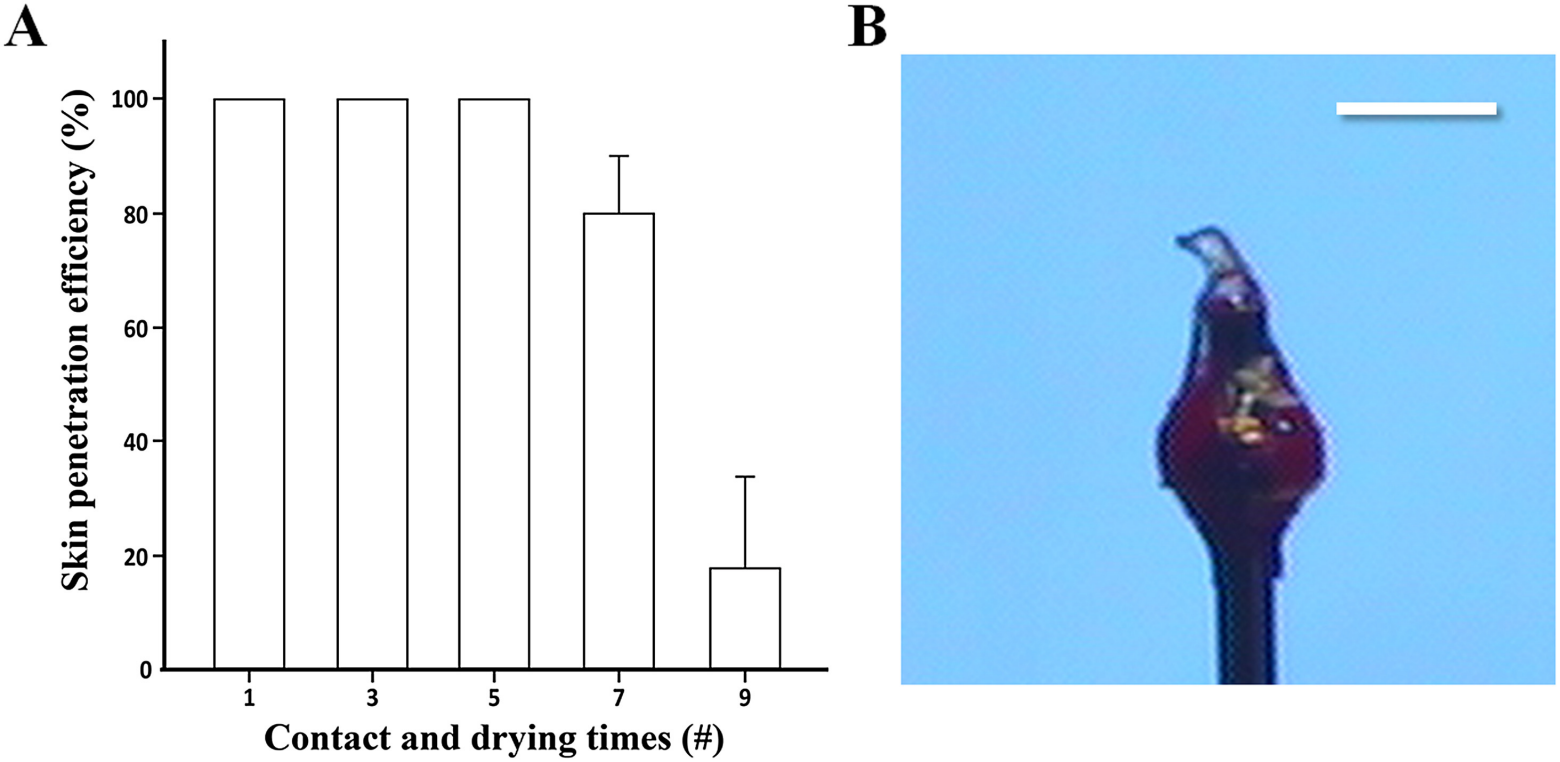
Evaluation of skin insertion failure. (A) DMNs created by 1 to 5 drying cycles completely penetrated the skin, but DMNs generated by 7 or more cycles did not due to the fact that the maximum axial diameter of the MNs was roughly 2.5 folds larger than the pillar after 7 contact and drying cycles (n = 4). Data are shown as mean ± s.d. (B) Skin insertion failure. Broken DMN on the pillar after application. Scale bars, 500 μm.

DMNs fabricated by 1, 3, and 5 contact and drying cycles showed 100% of skin insertion frequency. That means that all DMNs penetrated and inserted into skin without buckling or fracture of DMN structure. However, when more than 7 contact and drying cycles applied, the DMNs failed to insertion because their axial diameter was roughly 2.5 times higher than the diameter of the pillar (Fig 3A). When these over-sized DMNs were administered to the skin, they failed to penetrate and the pillars pierced the center of the DMN base, eventually destroying the DMNs (Fig 3B). Further development of DMN applicator device may enhance frequency and reproducibility of skin insertion. We defined the Troy MN as DMNs that simultaneously completed insertion and also rapidly separated from the pillars.

### DMN-separation efficiency

**Fig 4:**
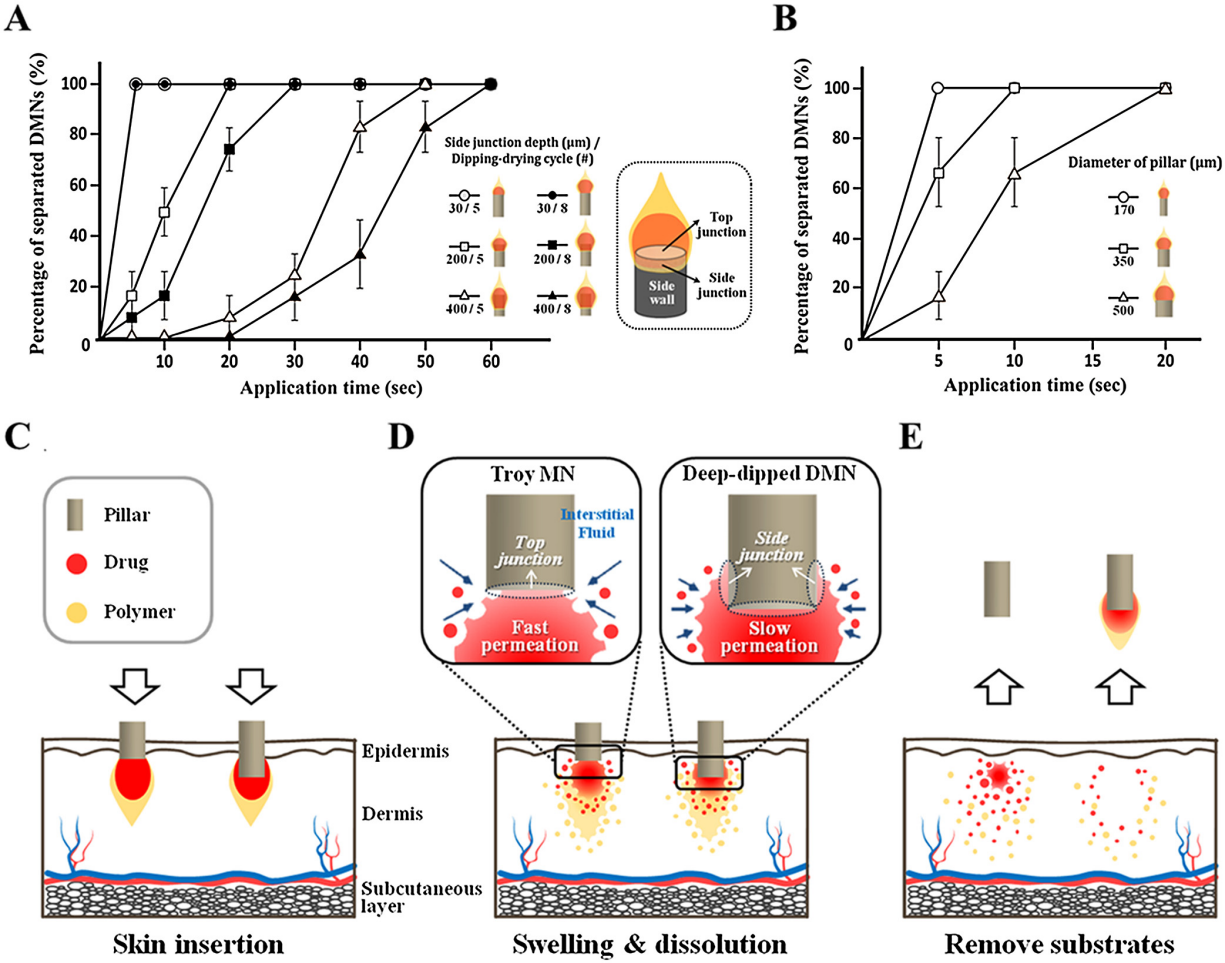
DMN-separation study with agarose gel and schematic illustration of DMN-separation mechanism. (A) DMN-separation efficiency according to side junction depth. Rhodamine B-encapsulated DMNs (1 × 3) were inserted into agarose gel and examined right after removing pillars at each designated application time. Side junction depths were set to 30 (contact, not immersion), 200 and 400 μm, and the number of contact (dipping) and drying cycles was set to 5 and 8 times for each depth. As the side junction depth and number of cycles increased, DMN-separation efficiency decreased. Troy system DMNs separated within 5 s due to their minimum binding shape, and DMN volume did not influence separation time (n = 4). Data are shown as mean ± s.e.m. (B) DMN-separation efficiency according to top junction area. Three pillar types with different diameters (170, 350 and 500 μm) were used for Troy MN fabrication, with a uniform side junction depth of 30 μm and 8 contact and drying cycles. Pillar diameter influenced DMN-separation time due to the larger top junction area as pillar diameter increased, necessitating more time for complete DMN-separation (n = 4). Data are shown as mean ± s.e.m. (C) Two different DMNs with different binding shapes between the polymer matrix and pillars were penetrated into the skin. The Troy MN (left) was made only on the end surface of the pillars, while the deep-dipped DMN (right) had a greater side junction area. (D) Interstitial fluid easily permeated to the top junction of the Troy MNs (left square), but the additional polymer matrix on the pillar side walls of the deep-dipped DMNs inhibited interstitial fluid permeation to the top junction (right square). (E) After a few seconds, Troy MNs rapidly separated from the pillar, while more separation time was required for deep-dipped DMNs.

As shown in Fig 4A, DMN-separation efficiency was analyzed using transparent agarose gel, varying the side junction depth (30 **±** 5, 200 **±** 25 and 400 **±** 40 μm) and the number of contact (or dipping) and drying cycles (5 and 8 times). When the pillars just contacted the polymer solution during CCDP, a slight side junction (30 **±** 5 μm) was generated on the side wall of the pillar. The separation time increased from 5 to 20, and then 50 s as the side junction depth increased from 30 to 200, and then 400 μm with 5 cycles (open symbol in Fig 4A). A similar trend in DMN-separation was observed with 8 cycles (closed symbol in Fig 4A), due to the greater polymer coating around the top junction as the dipping depth and cycle number increased. The additional polymer coating on the side walls of the pillars may retard the access of interstitial fluid to this protected top junction, requiring more separation time.

Pillar diameter also affected DMN-separation time (Fig 4B). As pillar diameter increased from 170 to 350, and then 500 μm, DMN-separation time increased from 5 to 10, and then 20 s at a fixed side junction depth of 30 μm. Although there was no difference in access of the interstitial fluid to this top junction due to the same side junction depth, the greater top junction surface area led to delayed separation of the DMNs. Live images of DMN-separation in agarose gel are shown in S2 and S3 Figs.

Minimizing pillar side junctions, which was dependent on the dipping depth, turned out to be critical for successful fabrication of Troy MNs. A shallow side junction depth (30 μm) with a large pillar diameter (500 μm) yielded faster DMN separation than a deep side junction depth (400 μm) with a small pillar diameter (170 μm), even though the total conjunction (calculating the top and side junction area of the pillar) of shallow-dipped DMNs (2.5 × 10^5^ μm^2^) was higher than deep-dipped DMNs (2.3 × 10^5^ μm^2^). This finding indicates that DMN-separation time can be decreased more efficiently if DMNs are only fabricated onto the end surface of pillars, minimizing side junctions. Fabrication of DMNs on the end surfaces of the pillar was critical to DMN separation from the pillars.

The major advantage of Troy MNs is rapid separation of the DMNs from the pillars as a solid form without a need to wait for complete dissolution. Fabrication of the DMNs on the end surface of the pillars through CCDP allowed rapid dissolution at the top junction. As shown in Fig 4C and 4D, after insertion of the Troy MNs, solidified polymer started to swelling and dissolution occurred as the surrounding interstitial fluid begin to permeate into the top junction. This process caused the pillars to separate from the DMNs and ultimately embed the DMNs into the skin (Fig 4E).

Access to the top junction area for interstitial fluid is important for the Troy MN system, and can be controlled by how DMNs are fabricated on the pillars. When DMNs are created by controlled shallow dipping (30 μm dipping depth), the interstitial fluid easily permeates the junction, allowing rapid simultaneous swelling and dissolution with skin insertion (left square in Fig 4D). In contrast, deep-dipping (over 100 μm dipping depth) caused polymer matrix to coat the side walls of the pillars. As a result, the top junction was covered by an additional polymer coating that interfered with permeation by the interstitial fluid (right square in Fig 4D). Under these circumstances, the DMNs are delivered inside the skin in a fully dissolved form, which is less effective.

We also compared DMN-separation to microneedles coated by repeated dipping and drying of drug on the wall of the shaft of microneedle, designed so that the drug will dissolve into the skin from the microneedle surface after insertion. Because the shaft of a solid microneedle is designed to penetrate the skin, coated microneedles usually have a cone-like shape, and dipping the entire shaft is inevitable during coating. The result was similar to the deep coating in the CCDP method, which induced more side junction on the pillars. Accordingly, rapid separation of the polymer from the shaft of the coated microneedles was difficult. As shown in S4 Fig, the separation time for coated microneedles was 30 and 75 s at a depth of 500 and 900 μm, respectively. A similar separation time was observed in CCDP-created microneedles at a depth of 400 μm, but separation time was only 5 s at a depth of 30 μm. Although a greater depth of penetration was critical for the delivery of the drug load in coated microneedles, a much shallower depth accomplished drug delivery in microneedles coated by CCDP, where the DMNs were confined to the end surfaces of the pillars.

### Complete DMN delivery

**Fig 5:**
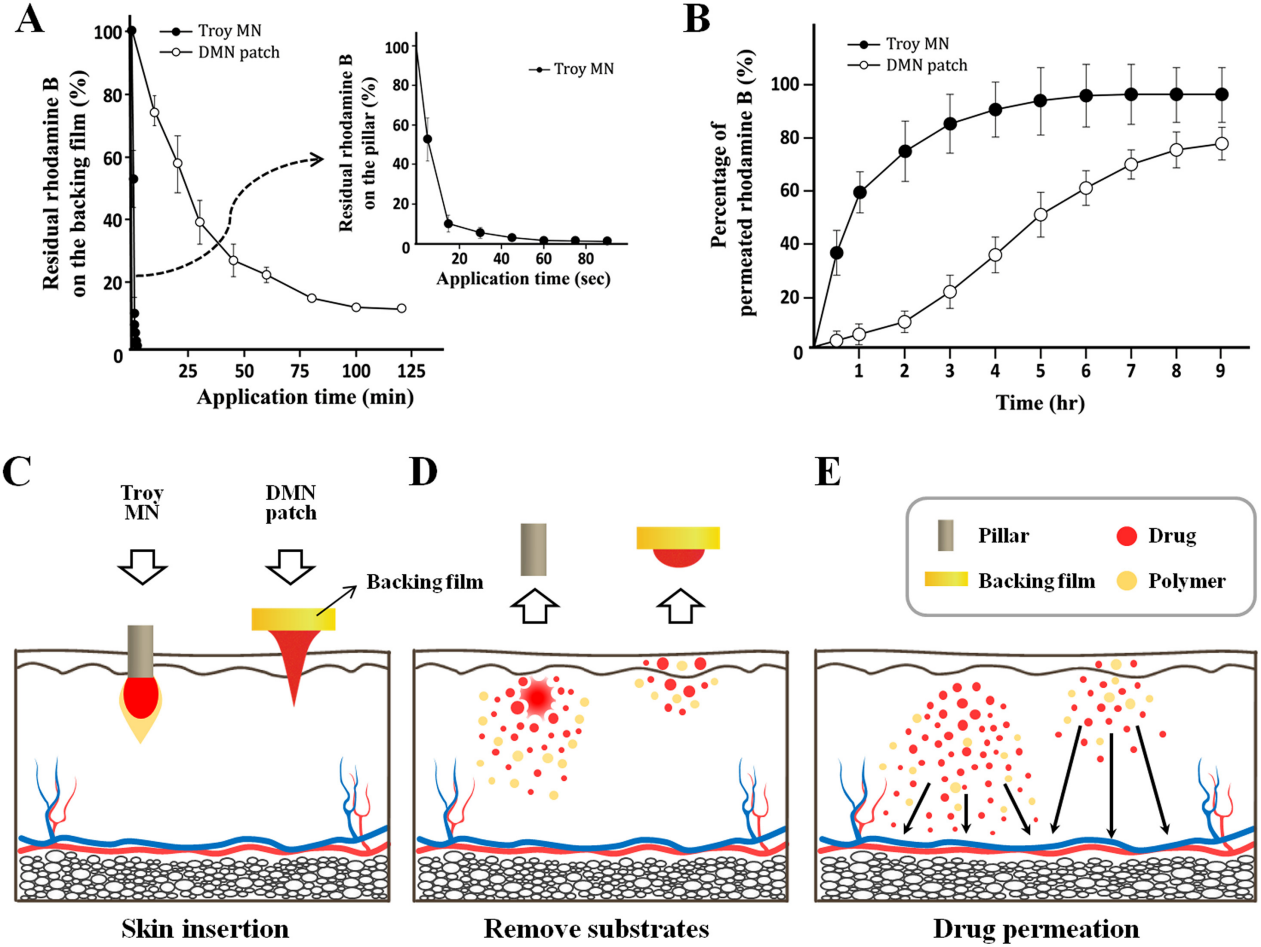
Comparative drug delivery efficiency study. (A) Drug delivery efficiency by controlled application time. With the Troy MN, more than 90% of the encapsulated rhodamine B separated from the pillar within 15s after application (dotted square). The DMN patch needed 500 times longer for 90% of encapsulated rhodamine B delivery. (B) *In vitro* skin permeation profile. The total amount of rhodamine B encapsulated in the Troy MNs rapidly diffused into the receptor chamber within 5 h. Diffusion kinetics were slower for the DMN patch, on the other hand, where 20% the encapsulated rhodamine B still had not diffused after 9 h (n = 3). Data are shown as mean ± s.d. (C-E) Schematic illustration of complete drug delivery and rapid diffusion of Troy MNs compared to a flat DMN patch. (C, D) The Troy MN completely penetrated into the skin, allowing the DMNs to be embedded in the skin. The traditional DMN patch, however, did not fully insert into the skin and residual drug remained on the backing film after application. (E) After application, Troy MNs yielded rapid drug permeability comparing to the DMN patch.

Before the permeability comparison study was carried out between Troy MNs and the DMN patch, we evaluated the delivery efficiency of encapsulated dye from the two substrates (pillar and backing film) by controlling application time (Fig 5A). In Troy MNs, more than 90% of the encapsulated rhodamine B separated from the pillars only 15 s after application, and 95% was separated after 30 s. In contrast, the DMN patch took much longer to deliver the dye into the skin, and 10% of the encapsulated rhodamine B remained on the backing film even 2 h after application. This difference implies that Troy MNs deliver encapsulated drugs 500 times faster than DMN patches. Because Troy MNs delivered more than 95% of encapsulated drugs within 30 s, no patch system was needed to hold the microneedles in place.

As shown in Fig 5B, Troy MNs achieved complete delivery and rapid drug permeability in an *in vitro* Franz cell experiment. Only 30 s after application, 97% of the encapsulated rhodamine B was completely delivered into the skin and rapidly diffused to the receptor chamber without remnant in the skin within 6 h. The DMN patch, on the other hand, retained 13% residual rhodamine B on the backing film even after 2 h on the skin, and 9% of the delivered rhodamine B remained in the skin after 9 h of diffusion due to slow permeability. Troy MN delivered larger amount of encapsulated drug than DMN patch at the given times (from 0.5 to 6 h after application, p < 0.01). In addition, Troy MN showed steady percentage of permeated rhodamine B from 7 h after application, and delivered amount of rhodamine B was significantly larger than DMN patch (p = 0.016). As the amount of encapsulated model drugs were equal (10 μg of rhodamine B), total amount of delivered model drug was certainly higher in case of Troy MN, compared to DMN patch. These results demonstrate that DMN fabrication by CCDP satisfies both goals of complete insertion and rapid administration of encapsulated drug.

In DMN-mediated therapy, delivery of an exact amount of drug is critical for the desired efficacy. As shown in Fig 5C and 5D, Troy MNs pierce the skin directly, overcoming the elasticity of skin, because of the sufficient mechanical force provided by the strong metal pillars. The pillars can penetrate just beneath the epidermis (roughly 100 μm penetration depth) with minimal invasion due to their micro-sized geometry (170 μm diameter). The DMNs can then be embedded in the skin, and the total surface of the polymer matrix can be exposed to sufficient interstitial fluid. In traditional DMN patches, on the other hand, the DMN array cannot be embedded into the skin because the microneedles are strongly bound to the flat backing film. The stiffness of the sticky patch fails to provide enough mechanical force, preventing the base of the DMNs from fully inserting into the skin and delivering the full dose of drug. Troy MNs retain no residual drug on the pillars, while traditional DMN patches maintain residual drug on the backing film. Complete insertion of the Troy MNs allowed accelerated drug permeation, facilitating rapid achievement of the target blood drug content (Fig 5E). Recent research has demonstrated that microneedle penetration depth is the most critical factor in determining drug permeability [16]. The DMN patch, however, delays drug permeation because the drugs are mostly initially deposited in the uppermost layers of skin.

### *In vivo* skin penetration study

**Fig 6:**
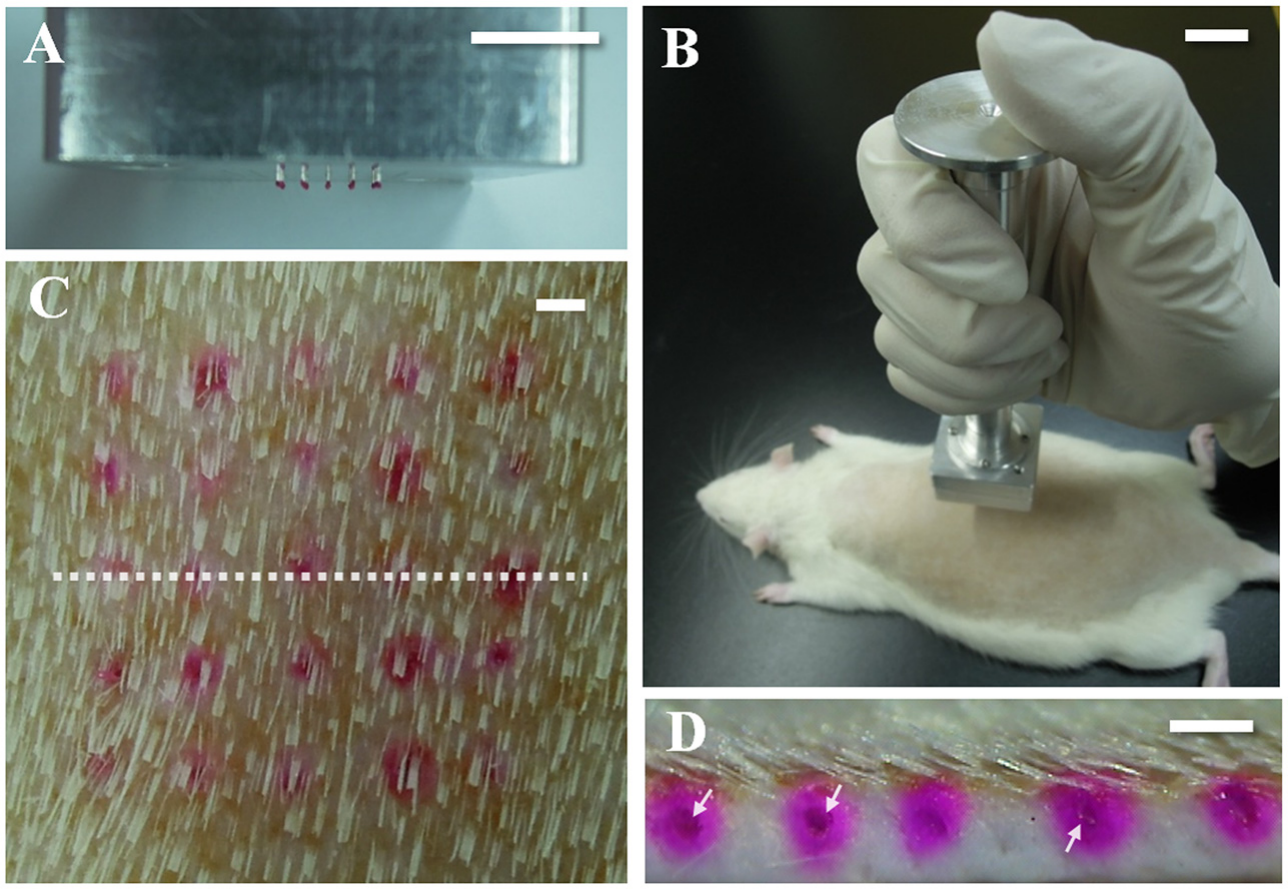
*In vivo* skin penetration study. (A) Troy MNs were assembled with an applicator into an array (5 × 5). (B) The applicator was applied to rat dorsal skin vertically by hand. (C) Image of skin with applied Troy MNs. The array of red spots indicates the penetrated site of rhodamine B-encapsulated Troy MNs and the white dotted line represents the vertically sliced line used to obtain sectional tissue. (D) Skin sectional image. Red spots mark delivered rhodamine B in the skin and the white arrow indicates undissolved parts of DMNs. Scale bars, 10 mm (A, B) and 1.0 mm (C, D).

As shown in Fig 6, rhodamine B-encapsulated Troy MNs (5 × 5) successfully penetrated into rat dorsal skin. The Troy MNs were assembled with an applicator and administered vertically to the rat dorsal skin (Fig 6A and 6B). Thirty seconds after application, the DMNs were fully separated from their pillars and embedded into the skin (Fig 6C and 6D). As shown in Fig 6D, the rhodamine B was concentrated in the center of the diffusion area (white arrow) and not fully dissolved yet. This result can be interpreted to that DMN tip may be in between intact and fully dissolved form; In detail, close to intact form because separation step is so rapidly finished compared to dissolution step. This demonstration supports the rapid and complete delivery of encapsulated drug to target site. However, there are several hurdles to overcome for practical application of Troy microneedle. First, polymer of high molecular weight, such as 360 kDa PVP used in this study, might cause accumulation in liver when it is applied frequently. Problem of bioaccumulation is an important issue which the entire field working on dissolving microneedle need to think about. Therefore, further study about bioaccumulation of polymer should be conducted. In addition, safety issue such as sterilization should be considered for drug and vaccine delivery, at level of practical application.

## Conclusions

Troy MNs, created via CCDP to consist of DMNs fabricated onto the end surface of pillars, enabled fast and complete administration of encapsulated drug. The Troy MN system overcomes critical challenges of the traditional DMN patch, including a long waiting period and low penetration efficiency. This novel DMN formation on pillars facilitated fast DMN-separation without waiting for dissolution of the polymer matrix, allowing complete delivery of the entire encapsulated drug into the skin by overcoming the viscoelastic skin barrier. In particular, the minimum binding between the DMNs and supporting materials was critical to DMN-separation efficiency. We expect the Troy MN to open a progressive route for DMN-mediated therapy by satisfying patient convenience with the desired drug efficacy.

## Supporting Information

S1 Fig DMN arrays on the flat patch. Rhodamine B-loaded DMNs (750 μm long) were fabricated on CMC backing film. Scale bars, 1.0 mm.
(TIF)

S2 Fig DMN-separation efficiency according to side junction depth. DMN-separation was evaluated in agarose gel by varying side junction depth from 30±5 (A) to 200±25 (B) and 400±40 μm (C) and the number of contact (or dipping) and drying cycles from 5 (upper images in each A, B, C) to 8 (bottom images in each A, B, C). Scale bars, 500 μm.
(TIF)

S3 Fig DMN-separation efficiency according to top junction area. Three pillar types with different diameters of 170 (A), 350 (B) and 500 μm (C) were used for Troy MN fabrication. Side junction depth and the number of contact and drying cycles were maintained at 30 μm and 8 times, respectively. Scale bars, 1.0 mm.
(TIF)

S4 Fig Live detection of polymer matrix separation using coated microneedles. (A) Rhodamine B-loaded PVP polymer was created on sharp-tipped solid microneedles with a dipping depth of 500 μm and 5 dipping and drying cycles, (B) a dipping depth of 500 μm and 8 dipping and drying cycles, and (C) a dipping depth of 900 μm with 8 dipping and drying cycles. Scale bars, 1.0 mm.
(TIF)

S1 Table Specification of troy MN. (TIF)
