# Supplementary figures and images for: The Troy Microneedle: A Rapidly Separating, Dissolving Microneedle Formed by Cyclic Contact and Drying on the Pillar (CCDP)
Source: PLoS One. 2015 Aug 26;10(8):e0136513. doi: 10.1371/journal.pone.0136513 (PMC4550382; doi:10.1371/journal.pone.0136513)

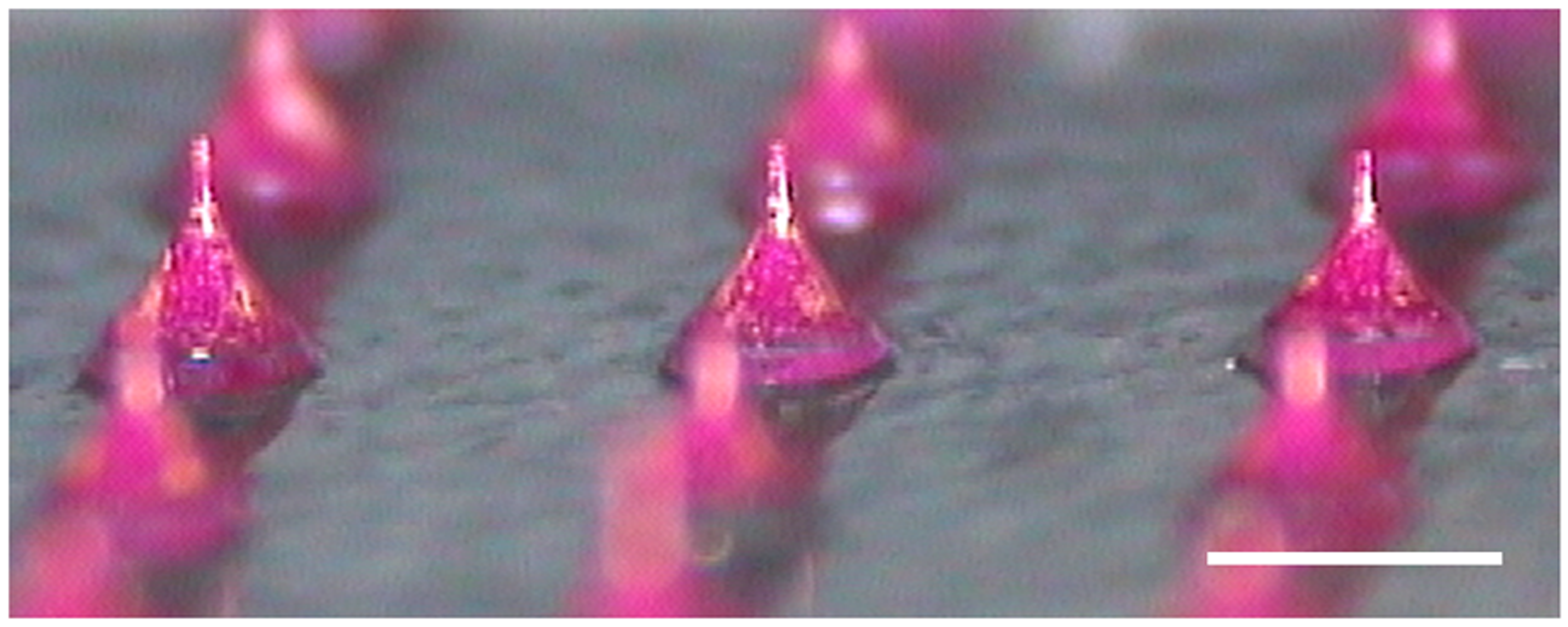

Supplement: S1 Fig — Rhodamine B-loaded DMNs (750 μm long) were fabricated on CMC backing film. Scale bars, 1.0 mm. (TIF) [file pone.0136513.s001.tif]

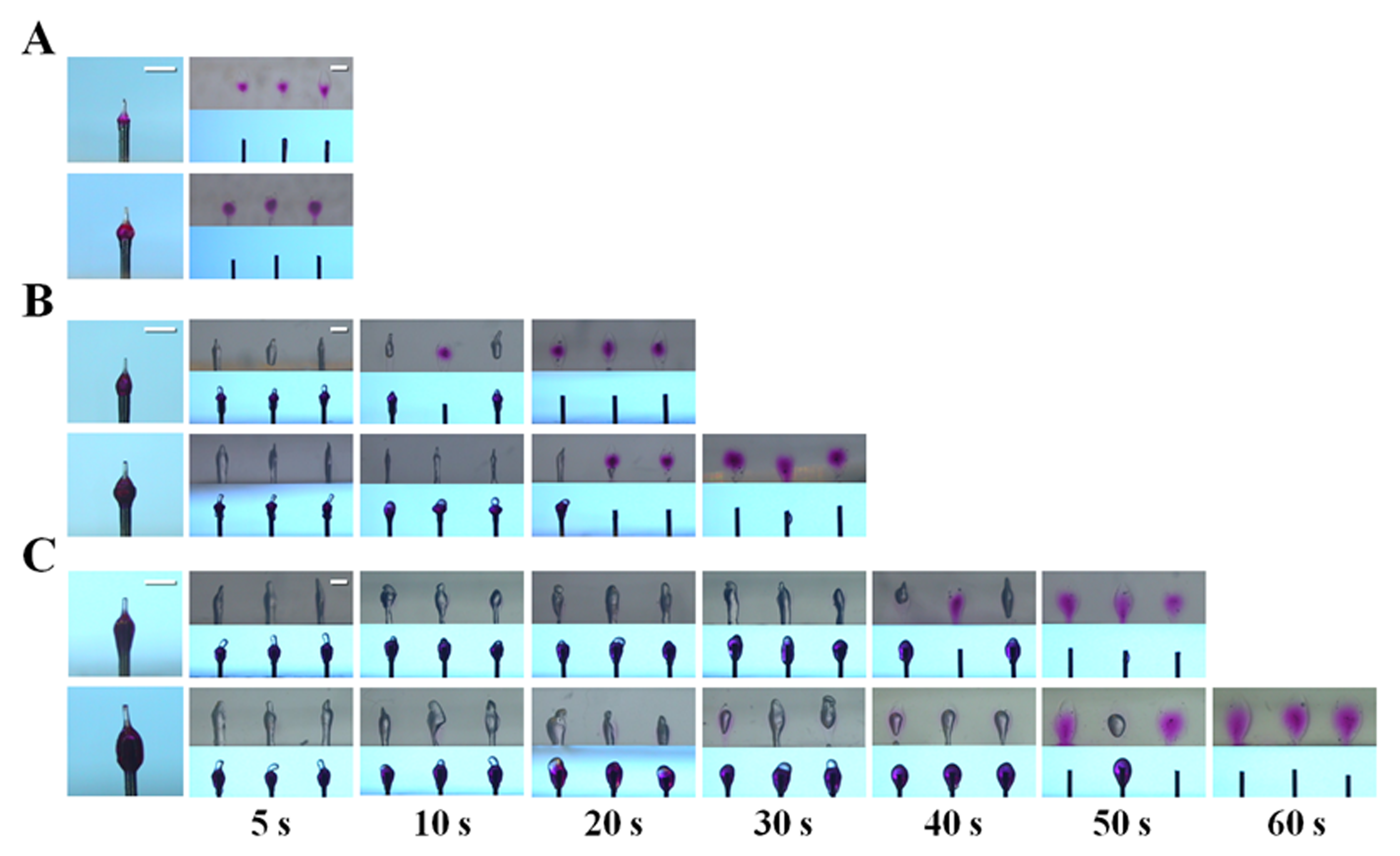

Supplement: S2 Fig — DMN-separation was evaluated in agarose gel by varying side junction depth from 30±5 (A) to 200±25 (B) and 400±40 μm (C) and the number of contact (or dipping) and drying cycles from 5 (upper images in each A, B, C) to 8 (bottom images in each A, B, C). Scale bars, 500 μm. (TIF) [file pone.0136513.s002.tif]

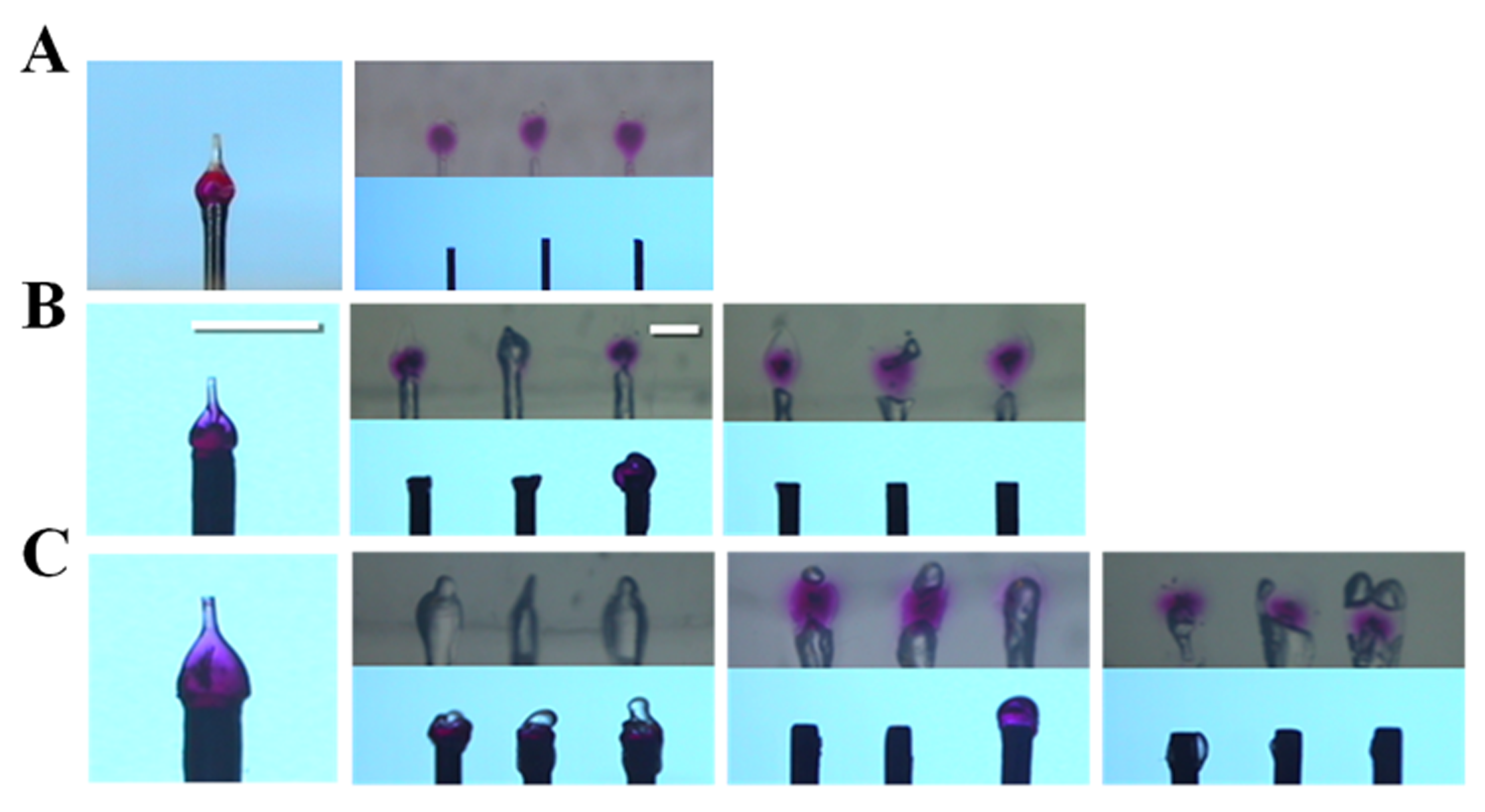

Supplement: S3 Fig — Three pillar types with different diameters of 170 (A), 350 (B) and 500 μm (C) were used for Troy MN fabrication. Side junction depth and the number of contact and drying cycles were maintained at 30 μm and 8 times, respectively. Scale bars, 1.0 mm. (TIF) [file pone.0136513.s003.tif]

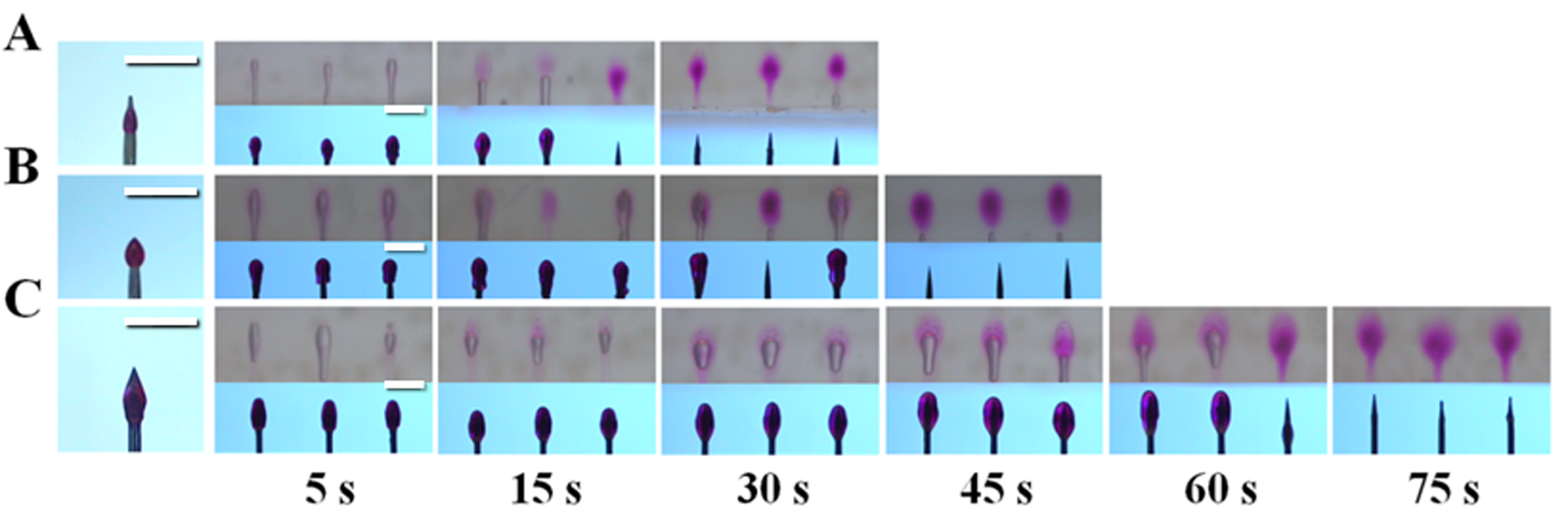

Supplement: S4 Fig — (A) Rhodamine B-loaded PVP polymer was created on sharp-tipped solid microneedles with a dipping depth of 500 μm and 5 dipping and drying cycles, (B) a dipping depth of 500 μm and 8 dipping and drying cycles, and (C) a dipping depth of 900 μm with 8 dipping and drying cycles. Scale bars, 1.0 mm. (TIF) [file pone.0136513.s004.tif]

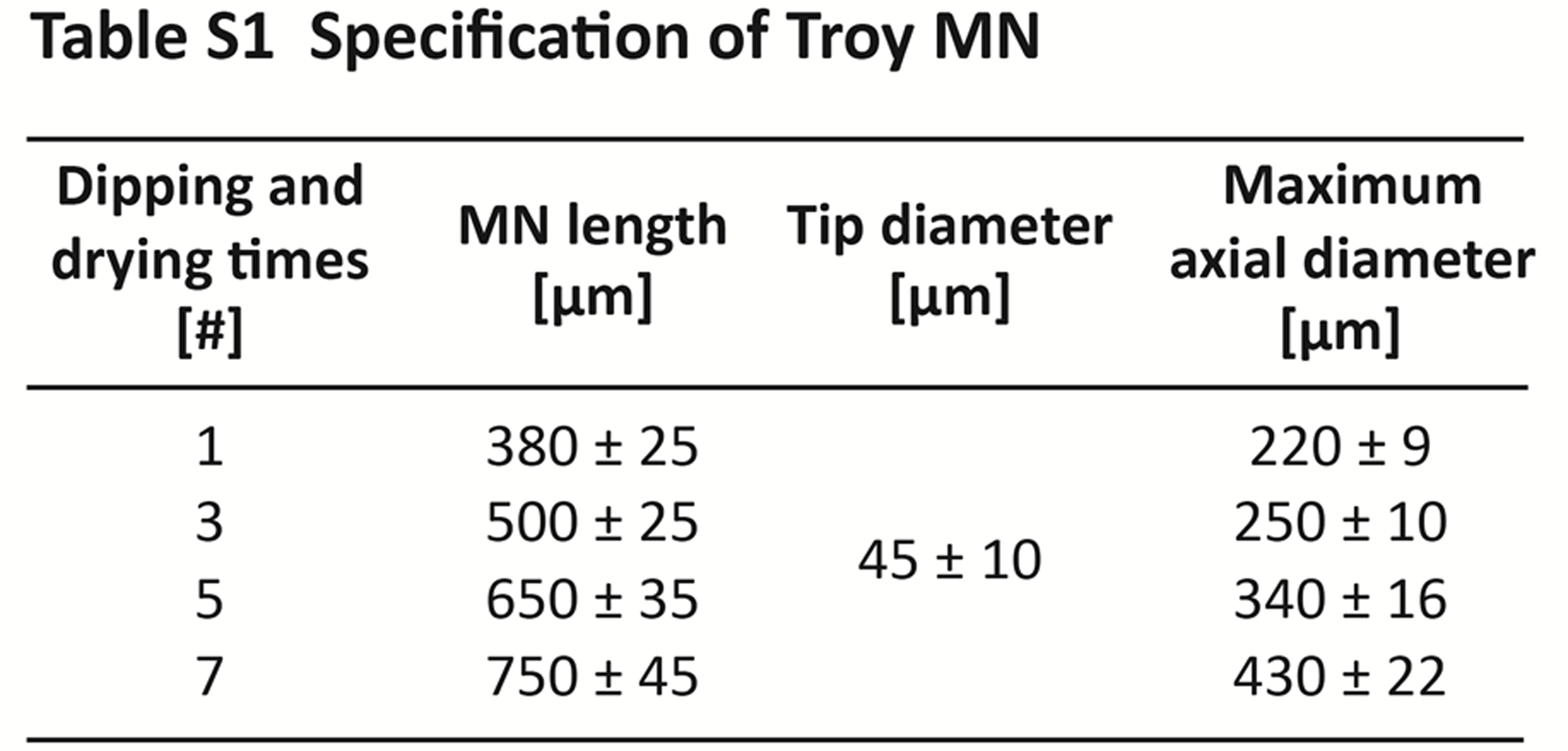

Supplement: S1 Table — (TIF) [file pone.0136513.s005.tif]
